# How shrub encroachment under climate change could threaten pollination services for alpine wildflowers: A case study using the alpine skypilot, *Polemonium viscosum*

**DOI:** 10.1002/ece3.3272

**Published:** 2017-07-28

**Authors:** Jessica A. Kettenbach, Nicole Miller‐Struttmann, Zoë Moffett, Candace Galen

**Affiliations:** ^1^ Department of Applied Ecology North Carolina State University Raleigh NC USA; ^2^ Biological Science Department Webster University Saint Louis MO USA; ^3^ Department of Biology Colorado College Colorado Springs CO USA; ^4^ Division of Biological Sciences University of Missouri Columbia MO USA

**Keywords:** climate change, heterospecific pollen transfer, plant–climate interactions, *Polemonium viscosum*, reproductive ecology, *Salix*, selection on floral traits, shrub encroachment

## Abstract

Under climate change, shrubs encroaching into high altitude plant communities disrupt ecosystem processes. Yet effects of encroachment on pollination mutualisms are poorly understood. Here, we probe potential fitness impacts of interference from encroaching *Salix* (willows) on pollination quality of the alpine skypilot, *Polemonium viscosum*. Overlap in flowering time of *Salix* and *Polemonium* is a precondition for interference and was surveyed in four extant and 25 historic contact zones. Pollinator sharing was ascertained from observations of willow pollen on bumble bees visiting *Polemonium* flowers and on *Polemonium* pistils. We probed fitness effects of pollinator sharing by measuring the correlation between *Salix* pollen contamination and seed set in naturally pollinated *Polemonium*. To ascertain whether *Salix* interference occurred during or after pollination, we compared seed set under natural pollination, conspecific pollen addition, and *Salix* pollen addition. In current and past contact zones *Polemonium* and *Salix* overlapped in flowering time. After accounting for variance in flowering date due to latitude, *Salix* and *Polemonium* showed similar advances in flowering under warmer summers. This trend supports the idea that sensitivity to temperature promotes reproductive synchrony in both species. *Salix* pollen is carried by bumble bees when visiting *Polemonium* flowers and accounts for up to 25% of the grains on *Polemonium* pistils. *Salix* contamination correlates with reduced seed set in nature and when applied experimentally. Postpollination processes likely mediate these deleterious effects as seed set in nature was not limited by pollen delivery. Synthesis: As willows move higher with climate change, we predict that they will drive postpollination interference, reducing the fitness benefits of pollinator visitation for *Polemonium* and selecting for traits that reduce pollinator sharing.

## 1 INTRODUCTION

**Figure 1:**
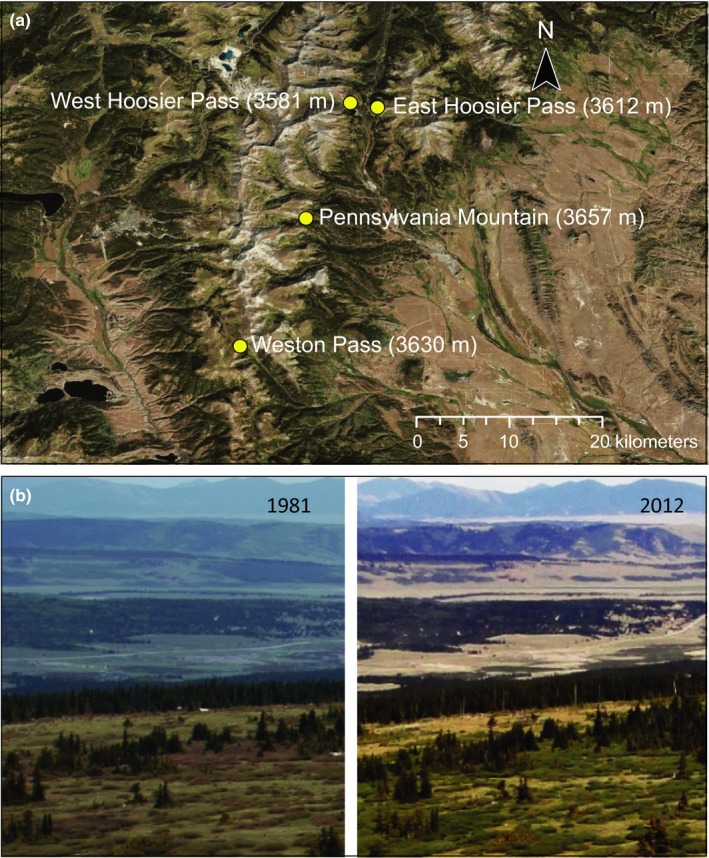
(a) Locations of four contact zones where surveys were conducted for *Polemonium* and *Salix* in the central Colorado Rocky Mountains (USA). (b) Photographs of the Pennsylvania Mountain contact zone in 1981 and 2012 showing infilling by encroaching *Salix* in alpine meadow habitat of *Polemonium*

Pervasive shrub encroachment into herb‐dominated ecosystems of high altitudes and latitudes can be attributed to climate change (Formica, Farrer, Ashton, & Suding, 2014; Huss et al., 2017). Warmer temperatures promote woody encroachment in alpine tundra ecosystems through infilling of openings and advancement of tree‐line (Myers‐Smith et al., 2011). Shrubs create living snow fences and a thick layer of leaf litter, impacting resident flora and fauna by altering microclimate and nutrient cycling (Becklin, Pallo, & Galen, 2012; Dona & Galen, 2007; Myers‐Smith et al., 2011). Paleo‐ecological evidence suggests that shrub species are preadapted to invade the open conditions of tundra biomes and have extended their ranges into high altitude and latitude ecosystems under past periods of favorable climate (Naito & Cairns, 2011). Opportunistic growth, clonal spread, and high allocation to reproduction increase biomass and cover (“shrubification”) for these woody gap species, allowing them to rapidly spread into meadow openings (Myers‐Smith et al., 2011; Fig. 1). These same life history traits have the potential to alter consumer behavior and reconfigure negative (herbivory) and positive (pollination) plant–animal interactions (e.g., Lara‐Romero, Garcia, Morente‐Lopez, Iriondo, & Brody, 2016; Muñoz, Celedon‐Neghme, Cavieres, & Arroyo, 2004). Here we ask whether encroachment of shrubby subalpine willows (*Salix glauca and brachycarpa*; hereafter *Salix*) into alpine ecosystems interferes with pollination and thus reduces fitness in a native alpine wildflower, the skypilot, *Polemonium viscosum* (hereafter, *Polemonium*).

In the Rocky Mountains of North America, willows along with other woody species are responding to warmer temperatures by expanding their altitudinal range and encroaching into herb‐dominated alpine vegetation (Formica et al., 2014; Petry et al., 2016; Fig 1). Willows are dioecious and plasticity in eco‐physiological traits of male shrubs and trees in particular, may promote invasiveness under climate change (Dudley & Galen, 2007; Petry et al., 2016; Tognetti, 2012). Petry et al. (2016) propose that broad environmental tolerance in males of dioecious species facilitates their upward migration. Male willows of the Rocky Mountain subalpine complex, *Salix glauca–brachycarpa* sustain shoot growth over a broad range of environmental conditions with extension of new shoots in one year giving rise to prolific flowering in the next (Dudley, 2006; Sakai, Sasa, & Sakai, 2006). Their massive floral displays and numerous, small pollen grains exemplify traits associated with negative heterospecific pollen receipt (hereafter, HP; Ashman & Arceo‐Gómez, 2013; Loughnan, Thomson, Ogilvie, & Gilbert, 2014; Carvalheiro et al., 2014).

Because willows are important early‐season flower resources for pollinators (Formica et al., 2014; Moquet, Mayer, Michez, Wathelet, & Jacquemart, 2015) altitudinal migration of males may also negatively affect pollination regimes of resident alpine wildflower species. At high altitudes and latitudes, *Salix* acts as a magnet species, drawing pollinators away from the more diminutive floral displays of tundra plants (Lara‐Romero et al., 2016; Mosquin & Martin, 1967). Here we investigate the potential for willow shrubification of alpine meadows to reduce reproductive success of resident wildflowers due to HP via pollinator sharing, focusing on the skypilot, *P. viscosum*, an obligate outcrosser that depends primarily on bumble bees for pollination (Galen, 1996).

The extent of pollinator sharing between insect‐pollinated species depends on their spatial proximity. *Polemonium* occurs over a 500 m altitudinal gradient from tree‐line to summit in the Rockies (Galen, Zimmer, & Newport, 1987). With altitude, this landscape decreases in surface area and thus habitable space (Elsen & Tingley, 2015). This geomorphological pattern implies that all else being equal, *Polemonium* populations will have broader ranges in lower parts of the alpine zone where shrubification is ongoing than on the upper tundra slopes that serve as refugia from this process. Separation of flowering times could moderate the impacts of spatial proximity in these low altitude contact zones (Poole & Rathcke, 1979). Flowering synchrony is promoted when species share environmental controls on timing of flower development (Post, Pedersen, Wilmers, & Forchhammer, 2008). Thus, if altitudinal immigrants converge with resident species in use of cues for budburst then competitive interactions may ensue among them (Brunet & Larson‐Rabin, 2012). Conversely, asynchrony may arise when close neighbors differ in cues for onset and tempo of flowering (CaraDonna, Iler, & Inouye, 2014; Galen & Stanton, 1995). Asynchronous flowering schedules are thought to evolve under a history of co‐occurrence, when costs of pollinator sharing exceed benefits. Here, we test for overlap in flowering times of *Salix* and *Polemonium* in contact zones and explore the potential role of shared sensitivity to warmer temperatures in promoting flowering overlap under climate change.

*Polemonium* is a model system for studies of pollinator‐mediated selection (Galen, 1996; Galen, Kaczorowski, Todd, Geib, & Raguso, 2011). If woody encroachment alters pollinator foraging behavior, it may select for functional traits that limit pollen interference before (e.g., corolla tube length; Galen & Cuba, 2001) or after pollination (e.g., style length; Ashman & Arceo‐Gómez, 2013). By elucidating timing of interference, experiments reported in this paper are a first step in addressing how pollinator sharing under willow encroachment may alter selection on these traits.

Specifically, we seek answers to the following questions:

1. Does flowering time overlap between *Salix* and *Polemonium* promote HP in contact zones?
2. Might convergent sensitivity to environmental cues for flowering synchronize *Salix* and *Polemonium* flowering schedules?
3. Do bumble bees that provide the bulk of pollination for *Polemonium* carry *Salix* pollen to skypilot flowers in contact zones?
4. Does *Salix* pollen reduce seed set in *Polemonium* through events during either pollen delivery or postpollination (pollen germination) phases?
5. How might encroachment of *Salix* HP into alpine habitats alter selection on reproductive traits in *Polemonium?*

## 2 MATERIALS AND METHODS

### 2.1 Study system

Interactions between willows, *Salix brachycarpa‐glauca*, and *P. viscosum* were studied from 1 June–31 July, 2015–2016 at four geographically isolated contact zones in the central Colorado Rocky Mountains (USA; Fig. 1a): Pennsylvania Mountain Natural Area (Park Co., 39.253539′N, 106.121690′W); Hoosier Pass West (Summit Co., 39.365065′N, 106.077243′W); Hoosier Pass East (Park Co., 39.35806958′N, 106.05191715′W), and Weston Pass (Park Co., 39.12718009′N, 106.18381408W). At all sites, plants were studied over an altitudinal range of 3,500–3,650 m. Vegetation ranged from a willow‐spruce mosaic (krummholz) at tree‐line to dry alpine meadows, ~50–100 m further upslope. *Polemonium* occurs in openings in the krummholz mosaic that have infilled due to willow encroachment over time (Fig. 1b). *Salix* and *Polemonium* flower early in the season, typically in mid to late June in these four low alpine contact zones. Both species provide early food resources for flower‐visiting insects in high altitude habitats and are heavily frequented by bumble bee queens during nest establishment (Byron 1978). Individuals depend on insect pollination for outcrossing and reproductive success. The small dioecious flowers of *Salix* bloom in a mass display, offering pollen (male flowers) and nectar (both sexes) to bee and fly pollinators. Plants of *Polemonium* have showy, nectar‐rich flowers and are pollinated primarily by long‐tongued nectar‐foraging bumble bees (especially queens of *Bombus balteatus;* Galen & Kevan, 1980; Galen, 1996).

### 2.2 Flowering phenology

We noted incidence of flowering time overlap in the four contact zones described above and surveyed past flowering records using herbarium specimens collected from 25 Colorado Rocky Mountain sites, between 1907 and 2009 to broaden our geographic sampling. Herbarium databases were hosted by the University of Colorado (Boulder, CO), Colorado State University (Fort Collins, CO), and the Rocky Mountain Herbarium (Univ. of Wyoming; Cheyenne, WY). Flowering status was obtained from labels, digital images of specimens, and visual examination for specimens housed at the Carter Herbarium of Colorado College (Colorado Springs). We searched all *Salix glauca*,* S. brachycarpa*, or *S. glauca‐brachycarpa* and *Polemonium viscosum* specimens in these four databases to identify locations above tree‐line (>3,300 m altitude) where both species were collected in flower during the same year. Twenty‐five sites met these criteria, with *Salix* and *Polemonium* occurring within a 200 m altitudinal range. A paired (by site) *t*‐test was used to test whether collection date of flowering specimens (Julian Day) differed between *Salix* and *Polemonium* across the range of conditions encompassed in this sample. To test whether flowering overlap of *Salix* and *Polemonium* may reflect a shared sensitivity to temperature at budburst, we performed a second analysis (ANCOVA *lme* in the *nlme* package (Pineiro et al. 2011) and ANOVA in the *car* package (Fox et al. 2013)) on specimen collection date with temperature (statewide average minimum temperature in June of the collection year (NOAA; Western Regional Climate Center), species and their interaction as fixed effects and spatial variation (latitude at the center of the county where collections were made) as a random effect. Statewide temperature data were used to distinguish warm seasons from cold ones because local data were not available for all sites and collection years. The proportion of variance explained by the fixed and random effects was calculated via *r.squaredGLMM* (*MuMIn* package (Bartoń and R Development Core Team 2015)). All statistical analyses were conducted using R statistical language (R Development Core Team 2016), unless otherwise noted.

### 2.3 Pollinator sharing

In 2016, we surveyed the frequency with which primary pollinators of *Polemonium*, (queens of the bumble bee species *Bombus balteatus*) carry *Salix* pollen when foraging from *Polemonium* flowers in contact zones. Ten bees (2–3 per site) were captured during visits to *Polemonium* within 20 m of *Salix* and cooled in vials to facilitate handling. To sample from the pollen pool available for deposition during flower visits, a small cube of fuchsin‐stained gelatin was dabbed gently on noncorbicular areas of the bee's head and body. Bees were then returned to the field and released. Gelatin cubes were melted onto microscope slides and scanned for *Salix* grains. Because sampling success was likely to vary among specimens due to differences in body size and activity, we scored slides for the presence, but not amount of *Salix* pollen.

To survey the amount of *Salix* pollen deposited on pistils of naturally pollinated *Polemonium* flowers, we collected styles from senescing flowers of thirty randomly chosen plants in each of the four sites surveyed in 2016. Plants were sampled within a range of 1–20 m from the nearest *Salix*. Styles were collected from 2 to 3 flowers per plant after the corollas began to wilt, mounted on slides and stained with fuchsin gel to facilitate pollen identification and enumeration in the laboratory. To assess the prevalence of willow HP at the regional scale and its spatial distribution within the contact zone, we conducted an analysis of covariance with the percentage of *Salix* pollen in the stigma pollen load (*Salix* contamination) square root transformed to conform to assumptions of ANCOVA as the dependent variable, and site and distance from the nearest *Salix* shrub as fixed effects (*lm* in the *stats* package; R Development Core Team 2016). Here and elsewhere we use *Salix* contamination as an index of HP to facilitate comparisons with the literature (Ashman & Arceo‐Gómez, 2013) and because heterospecific contamination has been demonstrated a major component of pollination quality in past studies of *Polemonium* (Galen & Gregory, 1989).

### 2.4 *Salix* pollen interference:

In 2015, we identified twenty spatially separated (≥5 m) patches of *Polemonium* in the contact zone on Pennsylvania Mountain and randomly selected two plants separated by ≥1 m in each patch to assay *Salix* HP and seed set under natural pollination. Plants were selected in bud and their flowers were protected from nectar‐thieving ants by surrounding the inflorescence stems with sections cut from plastic drinking straws and coated with Tanglefoot (Galen 1983). Plants had 11.5 ± 3.2 (*SD*,* n* = 40) flowers each. The style was removed from one flower and mounted in fuchsin gel to enumerate deposition of *Salix* and conspecific pollen. Prior research shows that most of the variance in pollen receipt among flowers of *Polemonium* is due to plant‐to‐plant differences rather than variation among a plant's flowers (Galen & Stanton, 1989). Stigmas withered and dropped from the flowers on eight inflorescences before collection, reducing the sample size for assessment of *Salix* HP to *n* = 32. After all flowers wilted, plants were protected from ungulate herbivores using hardware cloth cages. In September, we collected the infructescences and dissected the fruits for seed counts. Average seed set per flower was calculated as the total seed set per plant, divided by the total number of flowers, and was used as a measure of reproductive success. One infructescence was lost due to elk herbivory, reducing the sample size for seed set to *n* = 39.

We counted pollen on slides in the laboratory at the State University of New York at Old Westbury using light microscopy. All conspecific and *Salix* pollen grains under the coverslip were counted for each specimen. We computed a nonparametric (Spearman's) correlation between average seeds per flower and *Salix* contamination (SAS Proc CORR; 2013). Spearman's correlation was used because data did not fit assumptions of parametric statistics due to heteroscedacity, and transformation did not resolve the problem.

To probe the putative causal factors giving rise to associations between *Salix* HP and seed set in nature, we concurrently conducted two experimental pollination treatments. The first treatment, application of *Salix* pollen to all flowers on each plant at the onset of receptivity, tested whether the relationship between seed set and contamination in nature has a causal basis. In the second treatment, hand‐supplementation of outcross pollen to all flowers tested for pollen‐limitation of seed set in nature. If *Salix* HP decreases seed set in nature by blocking pollen delivery, then supplementing outcross pollen by hand should increase seed set relative to naturally pollinated controls. Conversely, a negligible impact of pollen addition would suggest that any negative effect of *Salix* HP in nature is mediated by postpollination processes.

In each of the twenty patches, all flowers on two additional randomly selected plants were pollinated with supplemental conspecific pollen (*n* = 40). In twelve of the experimental patches, all flowers on another randomly selected *Polemonium* plant were pollinated with *Salix* pollen. Pollen was applied by hand to all flowers of plants in conspecific and *Salix* pollen addition treatments on the first day of female receptivity (stigma lobes unclasped) Dehiscing anthers were taken respectively from flowers of a *Polemonium* plant at least 1 m away or from the closest male *Salix* and brushed over the receptive stigma lobes until a layer of pollen was visible. Calyces of the few flowers receptive before the onset of experimental pollinations were marked with a small dot of enamel paint and excluded from seed counts. After all flowers wilted, plants were protected from ungulate herbivores and seeds collections made as described for naturally pollinated individuals. We chose not to include a bagged outcrossed control because deleterious stigma clogging in *Polemonium* also occurs from pollinator‐mediated self‐pollination and HP from *Mertensia* and *Castilleja* (Galen & Gregory, 1989). Thus, excluding pollinators and generating a pure outcross “control” would eliminate other sources of stigma clogging and likely over‐estimate any negative impact of *Salix* HP.

Variation among treatments in seed set was analyzed using a mixed linear model (SAS Proc MIXED, 2013) with pollination treatment (conspecific pollen addition, *Salix* pollen addition, or open pollination) as a fixed effect and patch as a random effect. The average number of seeds per flower was square root transformed prior to the analysis to correct for heteroscedacity in the data.

## 3 RESULTS

### 3.1 Flowering phenology

**Figure 2:**
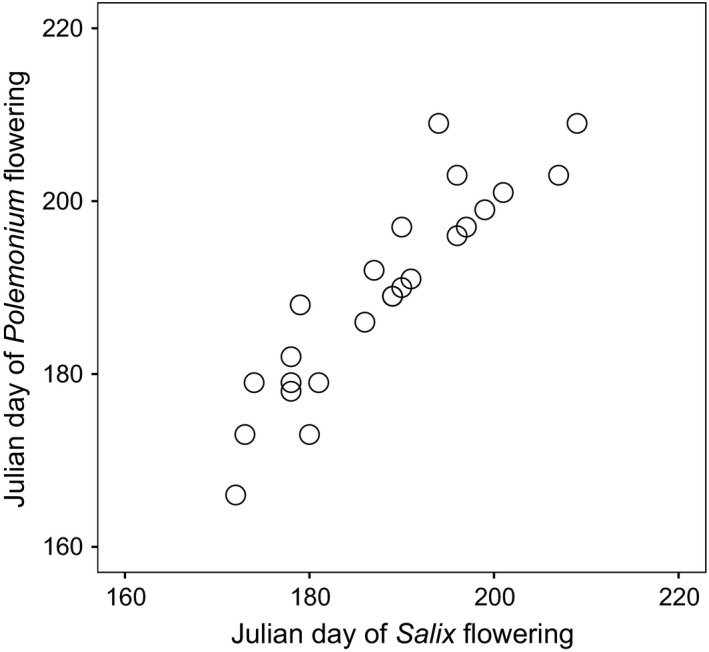
Relationship between Julian days when flowering specimens of *Salix* and *Polemonium* were collected in each of 25 historic contact zones in the Colorado Rocky Mountains (USA). Sites were sampled between 1907 and 2007 (*r* = 0.93, *df *= 23, *p* < .0001)

**Figure 3:**
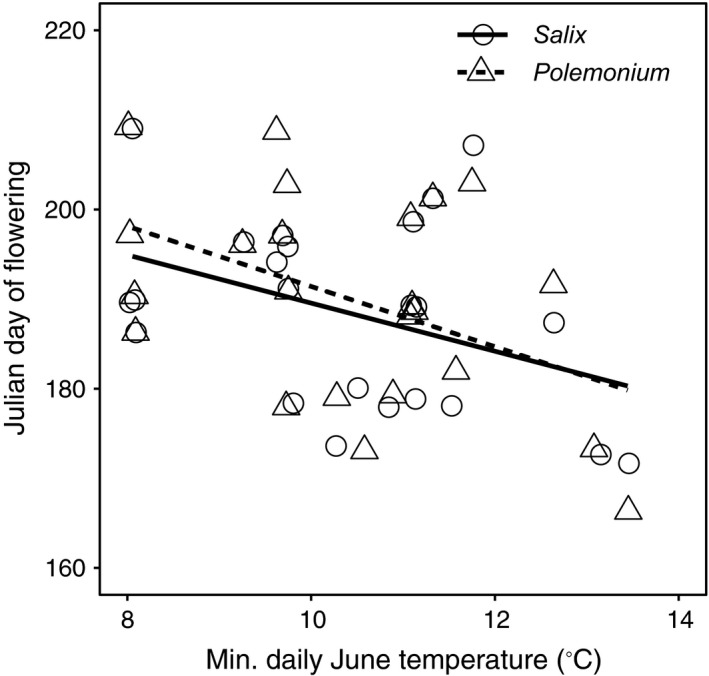
Julian day of collection for flowering *Polemonium* and *Salix* as a function of average daily low temperature in June of the collection year. Open circles and solid line show data for *Salix* while triangular symbols, and dashed line show data for *Polemonium* (*y* = 228.0−3.70*x* and *y* = 235.2−4.26*x*; respectively). Overlapping points are offset (*ggplot* with jitter width = 0.05 in R Statistical Software) in order to show all data points

*Salix* and *Polemonium* co‐flowered in all four extant contact zones surveyed in 2016. The 25 locations where collections allowed assessment of flowering time overlap in historic contact zones span a broad latitudinal transect of the Colorado Rocky Mountains, ranging from 37.5 to 40.5°N. Specimens of *Salix* and *Polemonium* were collected in 16 years between 1907 and 2009 at median altitudes of 3,505 m and 3,523 m, respectively. Statewide, minimum average temperatures early in the growing season (June) in years when collections were made ranged from 8.1 to 13.4°C. Based on collection dates (Julian Day) of these flowering specimens, we found no significant difference in flowering time of *Salix* versus *Polemonium* (paired *t*‐test, *t* = 1.45, *df* = 24, *p* = .16; Fig. 2). Analysis of covariance indicated that flowering times of *Salix* and *Polemonium* plants show similar advancement under warmer summers (Fig. 3). Latitude (random effect) accounted for 50% of the variance in flowering date and minimum average June temperature in the year of flowering (fixed effect) for 16% (χ^2^ = 5.579, *df = *1; *p* = .018). Average flowering dates (Julian dates) were similar between species (species χ^2^ = 0.2637, *df = *1; *p* = .607) and exhibited equivalent advancement in years of warmer temperatures (temperature by species interaction, χ^2^ = 0.169, *df = *1; *p* = .68; Fig. 3). Taken together, results indicate that co‐flowering of *Salix* and *Polemonium* occurred over a broad range of conditions reflecting similar sensitivity to temperature cues (or unmeasured environmental correlates of temperature) during budburst.

### 3.2 Pollinator sharing

**Figure 4:**
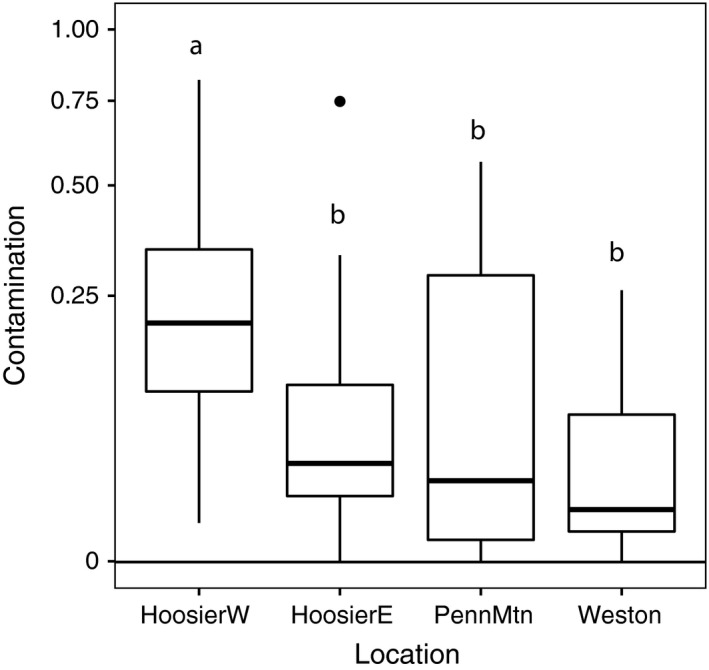
Variation among sites in contamination of *Polemonium* stigma pollen loads with *Salix* pollen (proportion of grains on the stigma belonging to *Salix*). Boxes show upper and lower quartile, and lines denote median values. Whiskers on each box indicate extremes, and solid circles denote outliers. Sites with different superscripts differ at *p* < .05

In 2016, all ten bumble bees (*Bombus balteatus* queens) captured while foraging on *Polemonium* flowers in four geographically isolated populations carried *Salix* pollen. *Polemonium* plants located near flowering *Salix* had stigma pollen loads with average contamination levels ranging from 4% to 25% among sites (*F* = 5.811, *df *= 3, *p* = .00099; Fig. 4). Proximity to willows had a negligible impact on contamination (distance *F* = 0.1355, *df *= 1, *p* = .71 regardless of site (for site by distance interaction, *F* = 1.3344, *df = *3, *p* = .2668).

### 3.3 *Salix* pollen interference:

**Figure 5:**
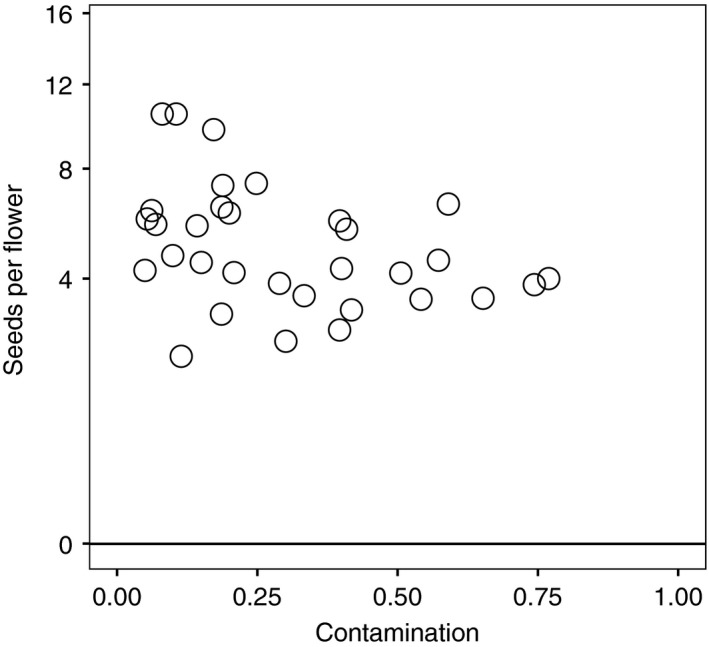
Relationship between contamination of *Polemonium* stigma pollen loads with *Salix* pollen and seeds per flower averaged for the plant. For the relationship, Spearman's *r* = −0.39, *df = *32, *p* = .0272

**Figure 6:**
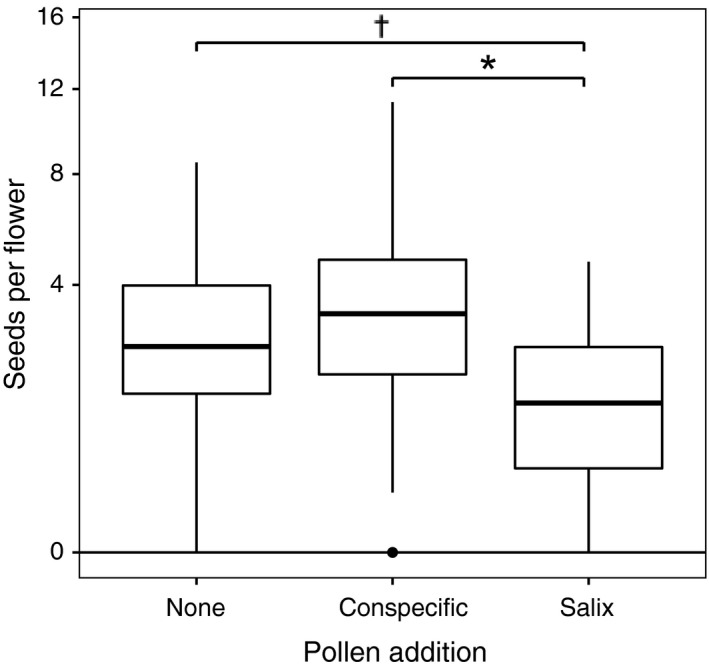
Seed set per flower for plants of *Polemonium* under natural pollination, conspecific pollen addition, and *Salix* pollen addition treatments. Boxes show upper and lower quartile, and lines denote median values. Whiskers on each box indicate extremes. Dagger indicates treatment difference at *p* < .07, and asterisk indicates difference at *p* < .05

*Salix* contamination showed a significant negative association with the average number of seeds per flower in naturally pollinated plants of *Polemonium* (Spearman's *r* = −.39, *df = *32, *p* = .0272; Fig. 5). Significant differences in seed set among experimental pollination treatments imply that this relationship has a causal basis (*F*_2,85_ = 3.35, *p* < .04). Plants subject to *Salix* pollen addition set significantly fewer seeds per flower than those with conspecific pollen supplementation (47% reduction; planned contrast, *t* = 2.58, *p* = .0117, Fig. 6) and tended to set fewer seeds per flower than open‐pollinated plants (*t* = 1.81, *p* = .0734; Fig 6). Seed set of open‐pollinated plants was similar to that of nearby individuals receiving supplemental conspecific pollen, indicating a lack of pollen‐limitation (Fig. 6) and suggesting that impacts of *Salix* contamination on fecundity were unlikely to reflect interference during pollen delivery.

## 4 DISCUSSION

Impacts of shrub encroachment on high altitude ecosystems have the potential to disrupt resident plant communities by changing resources including open space and light (Wilson & Nilsson, 2009); nutrient and water dynamics (Myers‐Smith et al., 2011); shelter and forage resources for mammalian and insect herbivores (Muñoz et al., 2004); and services of belowground (Becklin et al., 2012) and aboveground (Lara‐Romero et al., 2016) mutualists. Our results suggest that encroachment of willows can also reduce the fitness benefits of a pollination mutualism for the alpine wildflower, *P. viscosum*. Under a wide range of environmental conditions at low altitudes in alpine krummholz habitat, *Salix* co‐flowered with *Polemonium*. Convergence on temperature cues for budburst likely plays a role in flowering overlap, suggesting that pollinator sharing will continue as willows move upward onto the tundra with climate change. Because willows are magnet species for early‐season pollinators (Moquet et al., 2015), they attract a variety of insect pollinators including queens of the bumble bee species *Bombus balteatus* whose visits account for 60%–95% of pollination in *Polemonium* (Galen, 1996). Although these queens are relatively scarce and difficult to survey, *Salix* pollen was found on all ten individuals sampled during visits to *Polemonium* flowers and comprised up to a fourth of the pollen delivered to the stigmas. Contamination with *Salix* was associated with declining seed set in *Polemonium*. Experimental manipulation of pollen loads indicated that costs of *Salix* pollen precedence arise during postpollination stages of mating.

Phenological shifts due to changing temperature and snowmelt schedules under climate change can alter the nature and magnitude of pollinator sharing and thus generate indirect fitness effects on co‐flowering species. For example, advances in snowmelt under warming reduce overlap in flowering time, pollinator sharing, and potential competition for pollination in insect‐pollinated wildflowers of subalpine meadows (Forrest, Inouye, & Thomson, 2010). We surveyed flowering time overlap in four current contact zones and 25 locations where *Salix* and *Polemonium* co‐occurred during the past century. Over the range of environmental conditions encompassed in this survey, flowering dates for *Salix* and *Polemonium* did not differ significantly and indeed were highly correlated (*r* = .93; Fig. 2). While herbarium specimens may be subject to collector bias, direct observations of overlap in four geographically isolated contact zones support the conclusion of temporal overlap in flowering for these species. Together, results indicate that temporal overlap in flowering time is likely as *Salix* continues to encroach into the habitat of alpine *Polemonium*.

To elucidate potential mechanisms synchronizing flowering times of *Polemonium* and *Salix*, we examined flowering specimens of both species for evidence of advancement under warmer early‐season (June) temperatures. Because populations of willows and skypilots flower for up to a two‐week span (Galen & Kevan, 1980; Post et al., 2008), specimen collection date is a coarse indicator of flowering time. Nonetheless, data suggest that willows and skypilots show similar advancement in flowering period with warming (Fig. 3). Temperature is a driver of phenotypic plasticity in flowering schedule in wildflowers of high altitude environments and has also been reported to advance flowering in *Salix glauca* (Brunet & Larson‐Rabin, 2012; Inouye, 2008; Post et al., 2008). Our results are consistent with these findings though experimental temperature gradients are needed (e.g., Brunet & Larson‐Rabin, 2012) to eliminate confounding factors in alpine ecosystems (e.g., snowmelt, growing season length, and soil nutrient flux (Ernakovich et al., 2014).

Our sample size of queen *B. balteatus* was small, yet all ten individuals collected from *Polemonium* flowers carried *Salix* pollen. This finding aligns with the dwindling of historical floral resources for *B. balteatus*, a shift to generalized foraging and the evolution of shorter tongue length under climate change (Miller‐Struttmann et al., 2015). As *B. balteatus* queens represent the most effective pollinators for *Polemonium* (Galen, 1996), inclusion of *Salix* males in their foraging niche may generate especially deleterious HP effects. Subalpine willows have multiple features correlated with deleterious HP (reviewed by Arceo‐Gomez & Ashman, 2016). As obligate outcrossers, they produce massive floral displays and copious amounts of pollen. The environmental context of HP also appears to influence its severity. Alpine habitats of the central Rocky Mountains have nutrient poor soils and arid conditions exacerbating negative impacts of HP on reproductive success in other species (Celaya, Arceo‐Gómez, Alonso, & Parra‐Tabla, 2015).

For shrubby willows, the capacity for fast growth, broad environmental tolerance, and preadaptation to open environments may all contribute to their success as local invasives in high alpine ecosystems. Invasiveness predisposes donor species toward more negative HP impacts, perhaps because natives lack past opportunities to evolve tolerance or resistance (Arceo‐Gomez et al., 2016). This hypothesis explains the aggressive HP effects of exotic species, but the extent to which it applies to the locally invasive taxa undergoing “shrubification” is unknown. In light of our findings that willows have a strong negative HP impact on a common tundra wildflower, we suggest this idea deserves further attention.

While dioecy is not reported to correlate with HP globally, our results indicate that it may have a greater role than suggested by the limited studies to date. Because HP is intimately associated with male function, specialization of sexes to enhance mating success in dioecious species could intensify HP from male donors under pollinator sharing. For example, males typically exhibit advanced flowering schedules (Ågren, 1988; Esprito‐Santo et al., 2003; Lloyd & Webb, 1977) a feature that could amplify their attractiveness to insect pollinators early in the season when resources are few. Moreover, if, as Petry et al. (2016) propose, physiological plasticity in males confers an advantage during upward migration, dioecy could promote the rate of HP expansion into wildflower populations at high altitudes.

Comparison of seed production in plants with pollen‐supplemented flowers to that of controls indicated that pollinator visitation to *Polemonium* was not limiting in 2015. Thus, negative effects of *Salix* HP likely reflected postpollination processes. Clogging, by reducing contact of pollen pores with the proteins on the stigma surface, can restrict pollen germination and tube growth (Ashman & Arceo‐Gómez, 2013; Morales & Traveset, 2008) and in so doing, decrease the value of conspecific pollen delivery to flowers. Briggs et al. (2015)found similarly that HP interference in *Delphinium barbeyi*, weakened the relationship between pollen delivery and seed set. *Polemonium* populations harbor extensive phenotypic variation in functional traits including floral tube diameter, tube length, and volatile floral defenses (Galen & Cuba, 2001; Galen et al.,^1987^, ^2011^). Results of the present study suggest that as willows move upward, pollination interference may generate new selection pressures on reproductive traits that restrict *Salix* HP (e.g., stigma size and style length [Ashman & Arceo‐Gómez, 2013]).

In historically stressful alpine environments, plant recruitment is rare, and insect pollinators have a crucial role in promoting outcrossing and seedling establishment (Geib & Galen, 2012; Utelli & Roy, 2000). Our results indicate that the widespread “shrubification” of these high altitude ecosystems under climate change has potential negative indirect impacts on the quality of insect pollination for a widespread alpine wildflower. Given the implications of these effects for population recruitment, efforts to identify topographic conditions buffering *Polemonium* from willow HP are warranted. The rate of woody encroachment into alpine ecosystems shows well documented spatial trends, correlating with slope aspect across *Polemonium's* latitudinal range. These relationships suggest that such refugia from willow HP exist (Elliott & Cowell, 2015). Our results imply they may have considerable conservation value.

## AUTHORS CONTRIBUTIONS

JAK carried out field experiments, collected data, and contributed to the design of the study; NMS contributed to data analysis and presentation, writing and microscopy; ZM carried out herbarium and field surveys; and CG led the writing of the manuscript and design of the study. All authors contributed critically to the drafts and approved the final draft for submission to the journal.

## CONFLICT OF INTEREST

None declared.

## DATA ACCESSIBILITY

Data and SAS Code for all statistical analyses are archived at Dryad… Digital Repository. doi:10.5061/dryad.2p2bh
